# Correction: Prevalence of Active and Latent Yaws in the Solomon Islands 18 Months after Azithromycin Mass Drug Administration for Trachoma

**DOI:** 10.1371/journal.pntd.0006308

**Published:** 2018-03-07

**Authors:** The PLOS Neglected Tropical Diseases Staff

## Notice of republication

This article was republished on January 5, 2018, to correct errors with the supplementary data files during the typesetting process. The publisher apologizes for the errors. Please download this article again to view the correct version.

## Supporting information

S1 File Originally published, uncorrected article. (PDF)

S2 File Republished, corrected article. (PDF)
