# Supplementary material for: Correction: Prevalence of Active and Latent Yaws in the Solomon Islands 18 Months after Azithromycin Mass Drug Administration for Trachoma
Source: PLoS Negl Trop Dis. 2018 Mar 7;12(3):e0006308. doi: 10.1371/journal.pntd.0006308 (PMC5841639; doi:10.1371/journal.pntd.0006308)
Supplement: S2 File — (PDF) [file pntd.0006308.s002.pdf]

PLOS | NEGLECTED TROPICAL DISEASES

RESEARCH ARTICLE

# Prevalence of Active and Latent Yaws in the Solomon Islands 18 Months after Azithromycin Mass Drug Administration for Trachoma

**Michael Marks[1,2]*, Oliver Sokana[3], Eli Nachamkin[4], Elliot Puiahi[3], Georgina Kilua[5], Allan Pillay[4], Christian Bottomley[6], Anthony W. Solomon[1,2], David C. Mabey[1,2]**

**1** Clinical Research Department, Faculty of Infectious and Tropical Diseases, London School of Hygiene & Tropical Medicine, London, United Kingdom, **2** The Hospital for Tropical Diseases, London, United Kingdom, **3** Ministry of Health and Medical Services, Honiara, Solomon Islands, **4** Molecular Diagnostics & Typing Laboratory, Laboratory Reference & Research Branch, Division of STD Prevention, Centers for Disease Control and Prevention, Atlanta, Georgia, United States of America, **5** World Health Organization, Western Pacific Region Office, Honiara, Solomon Islands, **6** Department of Infectious Diseases Epidemiology, London School of Hygiene & Tropical Medicine, London, United Kingdom

* Michael.marks@lshtm.ac.uk

CrossMark
click for updates

OPEN ACCESS

**Citation:** Marks M, Sokana O, Nachamkin E, Puiahi E, Kilua G, Pillay A, et al. (2016) Prevalence of Active and Latent Yaws in the Solomon Islands 18 Months after Azithromycin Mass Drug Administration for Trachoma. PLoS Negl Trop Dis 10(8): e0004927. doi:10.1371/journal.pntd.0004927

**Editor:** Joseph M. Vinetz, University of California San Diego School of Medicine, UNITED STATES

**Received:** June 2, 2016

**Accepted:** July 25, 2016

**Published:** August 23, 2016

**Copyright:** This is an open access article, free of all copyright, and may be freely reproduced, distributed, transmitted, modified, built upon, or otherwise used by anyone for any lawful purpose. The work is made available under the Creative Commons CC0 public domain dedication.

**Data Availability Statement:** All relevant data are within the paper and its Supporting Information files.

**Funding:** MM is supported by a Wellcome Trust Clinical Research Fellowship–102807. AWS was supported by a Wellcome Trust Intermediate Clinical Fellowship–098521. Laboratory analyses of swab specimens was funded by the CDC. The funders had no role in design or conduct of the studies, the preparation of the manuscript or the decision to submit it for publication.

## Abstract

### Introduction

Both yaws and trachoma are endemic in the Pacific. Mass treatment with azithromycin is the mainstay of the WHO strategy for both the eradication of yaws and the elimination of trachoma as a public health problem, but the dose recommended for trachoma is lower than that for yaws. In countries where both diseases are endemic, there is a potential for synergy between yaws and trachoma control programs if mass treatment with the lower dose of azithromycin was shown to be effective for the treatment of yaws. In an earlier study, we demonstrated a profound reduction in the clinical and serological prevalence of yaws following a single round of mass treatment with azithromycin 20 mg/kg undertaken for the purposes of trachoma elimination.

### Methods

This survey was conducted 18 months following a single round of azithromycin mass treatment in the same communities in which we had conducted our previous six-month follow-up survey. We examined children aged 1–14 years and took blood and lesion samples for yaws diagnosis using the *Treponema pallidum* particle agglutination assay (TPPA) and the non-treponemal Rapid Plasma Reagin (RPR) test.

### Results

A total of 1,284 children were enrolled in the study. Amongst children aged 5–14 years, 223 had a positive TPPA (27.5%, 95% CI 13.6–47.7%). The TPPA seroprevalence amongst

**Competing Interests:** The authors have declared that no competing interests exist.

this age group did not differ significantly from either our pre-mass treatment survey or our initial follow-up survey. Thirty-five children had positive TPPA and positive RPR (4.3%, 95% CI 2.1–8.7%), and this did not differ significantly from our initial post-mass drug administration (MDA) follow-up survey (4.3% versus 3.5%, $p = 0.43$) but remained significantly lower than our initial pre-MDA survey (4.3% vs 21.7%, $p < 0.0001$). Village-level MDA coverage was strongly associated with dual-seropositivity ($p = 0.005$). Amongst children aged 1–4 years, 16 had a positive TPPA (3.5%, 95% CI 1.6–7.1%). This did not differ significantly from the seroprevalence in this age group that had been predicted based on our previous surveys (3.5% vs 5%, $p = 0.11$). Fourteen children (1.1%) were considered to have a skin lesion clinically consistent with yaws, but none of these individuals was seropositive for yaws. Of nine cases where a swab could be collected for PCR, all were negative for *Treponema pallidum* subsp. *pertenue* DNA.

## Discussion

In this study we have shown that the benefit of a single round of mass treatment with azithromycin 20mg/kg appears to extend to 18 months without any further intervention. The lack of a significant change in seroprevalence from 6 to 18 months after mass treatment might suggest that interventions could be spaced at yearly intervals without a significant loss of impact, and that this might facilitate integration of yaws eradication with other neglected tropical disease (NTD) control programmes. MDA coverage above 90% was associated with significantly better outcomes than coverages lower than this threshold, and strategies to improve coverage at all stages of yaws eradication efforts should be investigated.

## Author Summary

Yaws is a neglected tropical disease caused by a bacterium closely related to the agent of syphilis. Mass treatment is recommended by WHO for the control of both yaws and the blinding eye disease trachoma, but the dose used for trachoma is lower (20 mg/kg versus 30 mg/kg). We have previously shown that a single round of mass treatment with azithromycin for trachoma had a significant impact on the number of cases of yaws in a community, suggesting that the lower dose of azithromycin might be effective for yaws and that trachoma and yaws programmes in the Pacific might be integrated. We repeated our survey 18 months following the initial round of mass treatment to see if the benefit seen at 6 months had persisted. In this study, the number of yaws cases remained significantly lower than before mass treatment even without any additional public health interventions taking place in the 18 months between MDA and this follow-up. This might suggest that annual mass treatment could be used rather than the current recommendation of six monthly treatment. An annual treatment strategy might facilitate integrating yaws eradication efforts with other NTD control programmes.

## Introduction

Yaws is an endemic treponemal disease caused by *Treponema pallidum* subsp *pertenue* [1]. Most cases of yaws are seen in rural communities in tropical countries [2] and are manifest as lesions of the skin, bones and joints. In 2012, single dose azithromycin was shown to be an

effective treatment for yaws [3], and mass treatment with azithromycin was subsequently adopted by WHO as the cornerstone of a new yaws eradication campaign [4]. Active yaws is usually seen in children aged 5–14 years, and examination of this age group has been used in many studies to assess the prevalence of active yaws [5–7]. WHO also recommends surveillance by serology of children aged 1–4 years to determine whether transmission has been interrupted [4].

The Pacific is a particular focus for yaws with a large number of cases reported in Papua New Guinea, the Solomon Islands and Vanuatu [2]. Trachoma, caused by ocular infection with *Chlamydia trachomatis*, is also endemic in the Pacific. As community mass treatment with azithromycin is also central to the control of trachoma [8], there is the potential for synergies between trachoma and yaws programmes in the Pacific [9]. A challenge is the difference in the dose of azithromycin recommended for trachoma (20mg/kg, max 1g) compared to yaws (30mg/kg, max 2g). We previously demonstrated that the prevalence of active and latent yaws declined markedly six months after a single round of mass treatment with azithromycin given as part of a trachoma control program in the Solomon Islands [10], providing the first evidence that low dose azithromycin is an effective intervention to reduce the community prevalence of yaws.

The optimal number and timing of rounds of mass treatment needed to interrupt transmission of yaws are unknown [11], although pilot data from other countries suggest that a single round is inadequate [7]. In 2015 a decision was made not to undertake a second round of azithromycin mass treatment in the Solomon Islands in light of a low reported prevalence of trachomatous trichiasis and a low prevalence of ocular infection with *C. trachomatis* [12]. This provided an opportunity to assess any potential rebound in active and latent yaws in the absence of further control measures. It was hoped that these data might inform expansion of yaws control efforts and, in particular, any potential for synergy between yaws and trachoma control programs in the Pacific. We therefore conducted an 18 month follow-up survey to assess whether the seroprevalence of yaws has changed significantly in the absence of any further intervention.

## Methods

This survey was conducted 18 months following a single round of azithromycin mass treatment (20mg/kg) conducted by the Solomon Islands Ministry of Health and Medical Services. The study was conducted in the same communities in which we had conducted our previous six month follow-up survey [10]. No further rounds of mass treatment (or any other specific interventions against yaws including case finding surveys and targeted total treatment) had been conducted in these communities since then.

For each household we collected data on the number of residents. We enrolled children aged 1–14 years for assessment, collecting individual level data on age, gender, the presence or absence of clinical signs and symptoms of yaws and yaws treatment history. We categorized skin lesions using the WHO yaws pictorial guide [13]. All data were entered directly into Android smartphones using the ODK software package [14].

For children aged 5–14 years, venepuncture was performed and a serum sample collected. In children aged 1–4 years a finger-prick blood sample was collected onto a filter paper. Filter papers were air-dried and stored in sealed bags with desiccant sachets. From individuals with ulcerative or papillomatous skin lesions, we also collected a swab sample of lesion exudate. Exudate was transferred to a FTA Elute Micro Card (GE Healthcare, Buckinghamshire, UK) using three firm side-to-side motions of the swab across the card. Each card was placed in its own re-sealable plastic packet with an individual desiccant sachet. The samples were

transferred to the National Referral Hospital in Honiara, where they were frozen at -20°C, and shipped to the London School of Hygiene & Tropical Medicine (LSHTM), UK, and the Centers for Disease Control and Prevention (CDC), USA, on dry-ice for testing.

## Laboratory Testing

Blood samples were tested at LSHTM. Filter paper samples were eluted and tested using the *Treponema pallidum* particle agglutination test (TPPA, Mast Diagnostics, Merseyside UK) as previously described [15]. For serum samples a TPPA test was performed initially and for samples that were TPPA-positive, a quantitative rapid plasma reagin test (RPR, Deben Diagnostics, Ipswich, UK) was performed.

Lesion swab samples were tested at the CDC using a number of multiplex real-time (RT) PCR assays. Initially samples were tested using an assay for the identification of *T. pallidum* subspecies DNA [5]. If the PCR was positive for *T. pallidum* subsp. *pertenue* this was followed by a second multiplex RT PCR to detect mutations in the 23S rRNA gene which are associated with azithromycin resistance. All samples were also tested with a duplex RT PCR for the detection of *Haemophilus ducreyi* and *Mycobacterium ulcerans* [16]. All laboratory testing was performed by individuals masked to the clinical findings.

## Statistical Analysis

In children aged 1–4 years, a positive TPPA was taken as evidence of previous or current yaws infection. For children aged 5–14 years, a positive TPPA was considered as evidence of previous or current yaws infection. We considered individuals with clinical signs of yaws and both a positive TPPA and an RPR titre of ≥1:4 (dual-seropositivity) to have active yaws. We considered individuals who were dual-seropositive but without clinical signs of yaws to have latent yaws. For the purpose of the analysis an RPR titre of ≥1:16 was considered to be a high-titre positive. We classified household size as ≤5 or >5 residents, 5 householders being the national average according to the most recent census [17]. For each community, we used village level estimates of treatment coverage obtained during our previous six month follow up survey [10]. We classified village level coverage as low (<80%), high (80–90%) or very high (>90%). We calculated the prevalence of latent and active yaws in older children (aged 5–14 years) and the sero-prevalence of exposure to yaws in younger children (aged 1–4 years). Logistic regression was used to estimate unadjusted and adjusted odds ratios (ORs) for factors associated with both TPPA- and dual-seropositivity. Robust standard errors were used to calculate all confidence intervals (CIs) and p-values, to account for village-level clustering. All analyses were performed using Stata 13.1 (Statacorp, Texas).

## Sample Size

In our previous follow-up survey, the seroprevalence of yaws infection was 3.6%. We calculated that a sample size of 738 was need to measure a seroprevalence of 4% with a degree of absolute precision of 3%, assuming a design effect of 4.5 (estimated using data from the baseline survey) in children aged 5–14 years. Based on previous pre-MDA data from 5–14 year old children we hypothesized that the seroprevalence amongst children aged 1–4 years would be approximately 1% if transmission had been interrupted following the earlier community mass treatment and 5% if transmission had not been interrupted. We calculated that a sample size of 432 children aged 1–4 years was required to detect a prevalence of 1% with a precision of 2% and a design effect of 4.

## Ethical Approval

Written informed consent was obtained from each participating child's parent or guardian by a member of staff fluent in the local dialect. Assent was obtained from children. Ethical approval for the study was granted by the ethics committees of the Solomon Islands MHMS, the CDC, and LSHTM.

# Results

A total of 1,284 children were enrolled from 519 households in 10 communities.

811 children were aged 5–14 years (median 9 years), of whom 401 (49.4%) were male (Table 1). A serum sample was collected from 770 children (94.9%). Thirty-one children (3.8%) declined collection of a serum sample but assented to collection of a dried-blood spot, whilst 10 children (1.2%) assented to examination but not to sample collection. 473 children aged 1–4 years (median 3 years) were also enrolled, of whom 248 (52.4%) were male. A dried blood spot was collected from 451 children (95.3%) in this age group. Seven children (1.5%) aged under 5 incorrectly had a serum sample (rather than a DBS) collected. No dried blood spot was collected from 15 children aged under 5 (3.2%).

Four hundred and thirty seven children (34.0%) had at least one skin lesion. 14 children (1.1%) had lesions considered to be clinically consistent with yaws. The most common non-yaws lesions were scabies and impetigo, as previously reported (A Steer, Personal Communication). As in our initial 6 month follow-up survey, no individual with a skin lesion consistent with yaws had dual-positive serology. Swabs were obtained from nine yaws-like lesions. Swabs could not be obtained from the other three lesions as they were dry/crusted. Of these lesion samples, all were negative on RT-PCR for *T.p* subsp *pertenue* and two were positive for *H. ducreyi*.

Amongst 5–14 year-old children, 223 had a positive TPPA (27.5%, 95% CI 13.6–47.7%). The TPPA seroprevalence amongst this age group did not differ significantly from either our pre-MDA survey or our initial follow-up survey (31.4% and 25.0% respectively, $p>0.05$ for both comparisons). In both the crude and adjusted analyses, only age was associated with TPPA positivity (Table 2). Thirty-five children had dual positive serology (4.3%, 95% CI 2.1–8.7%), of whom 8 had high-titre positive serology. This did not differ significantly from our initial post-MDA follow-up survey (4.3% vs 3.5%, $p = 0.43$) but remained significantly lower than our initial pre-MDA survey (4.3% vs 21.7%, $p < 0.0001$). The level of MDA coverage was strongly associated with dual-seropositivity ($p = 0.005$). Compared to an MDA coverage above 90%, a village level coverage below 80% was strongly associated with an increased risk of dual-seropositivity (aOR 6.95, 95% CI 1.2–38.3, $p = 0.03$) (Table 3). Coverage of between 80–90% was also associated with an increased risk of dual-seropositivity compared to coverage >90%, but this difference was not statistically significant (aOR 3.99, 95% CI 0.66–24.0, $p = 0.12$).

**Table 1. Demographics of study subjects.**

| | | |
|---|---|---|
| **Number of Children** | **1284** | |
| **Number of Households** | 519 | |
| **Household Size [Number of residents] (Median, IQR)** | 6 (4–8) | |
| **Age [years] (Median, IQR)** | 6 (3–10) | |
| **Village Level azithromycin Coverage** | Low (<80%) | 536 (41.7%) |
| | High (80–90%) | 501 (39.0%) |
| | Very High (>90%) | 247 (19.2%) |

doi:10.1371/journal.pntd.0004927.t001

**Table 2. Risk factors for TPPA Positivity amongst children aged 5–14 years.**

| Variable | Indicative Prevalence Data | Unadjusted Odds Ratio (95% CI) | Adjusted Odds Ratio[#] (95% CI) | p-value |
|---|---|---|---|---|
| **Age [years]** | 5-9: 19% | 1.22 * (1.13-1.32) | 1.22 (1.13-1.32) | <0.001 |
| | 10-14: 38.5% | | | |
| **Male** | Male: 28.4% | 1.09 (0.77-1.57) | 1.19 (0.87-1.63) | 0.24 |
| | Female: 26.6% | | | |
| **Household Size [number of residents]** | ≤5: 30.21% | 1 | | |
| | >5: 25.6% | 0.8 (0.48-1.31) | 0.75 (0.46-1.23) | 0.23 |
| **Village Level azithromycin coverage[¶]** | Low Coverage (<80%): 24.1% | 1.13 (0.23-5.48) | 1.28 (0.26-6.39) | 0.73 |
| | High Coverage (80-90%): 33.4% | 1.83 (0.34-10.09) | 1.96 (0.31-12.56) | 0.44 |
| | Very High Coverage (>90%): 21.9% | 1 | | |

* Increased odds associated with each one year increase in age

# Adjusted for age and gender

¶ Compared to very high coverage (>90%)

doi:10.1371/journal.pntd.0004927.t002

Amongst children aged 1–4 years, 16 had a positive TPPA (3.5%, 95% CI 1.6–7.1%) including two children aged less than two years (0.9%, 95% CI 0.2–4.0%). This did not differ significantly from the seroprevalence in this age group that had been predicted based on our previous surveys (3.5% vs 5%, p = 0.11). In villages where very high (>90%) mass treatment coverage was achieved, there were no young children with a positive TPPA, compared to 3.8% and 4.4% TPPA positivity in villages with low (<80%) and high (80–90%) coverage of mass treatment, respectively, although these differences were not statistically significant (p = 0.08 and 0.06 respectively). Age was the only variable significantly associated with TPPA seropositivity amongst children aged 1–4 years (Table 4).

## Discussion

We have previously demonstrated that a single round of mass treatment with azithromycin at 20mg/kg, given for the purposes of trachoma control, has a significant impact on the prevalence of yaws-like skin lesions and sero-positivity for yaws [10]. In this study we have shown

**Table 3. Risk factors for Dual-Seropositivity amongst children aged 5–14 years.**

| Variable | Indicative Prevalence Data | Unadjusted Odds Ratio (95% CI) | Adjusted Odds Ratio[#] (95% CI) | p-value |
|---|---|---|---|---|
| **Age [years]** | 5-9: 3.3% | 1.18* (1.05-1.34) | 1.19 (1.08-1.35) | 0.009 |
| | 10-14: 5.7% | | | |
| **Male** | Male: 3.2% | 1.77 (0.85-3.71) | 1.91 (0.93-3.94) | 0.07 |
| | Female: 5.5% | | | |
| **Household Size [number of residents]** | ≤5: 5.7% | 1 | | |
| | >5: 3.3% | 0.57 (0.14-2.32) | 0.53 (0.13-2.14) | 0.33 |
| **Village Level azithromycin coverage[¶]** | Low Coverage (<80%): 6.2% | 5.5 (0.97-31.22) | 6.95 (1.27-38.3) | 0.03 |
| | High Coverage (80-90%): 4.1% | 3.6 (0.58-21.67) | 3.99 (0.66-24.0) | 0.12 |
| | Very High Coverage (>90%): 1.2% | 1 | | |

* Increased odds associated with each one year increase in age

# Adjusted for age and gender

¶ Compared to very high coverage (>90%)

doi:10.1371/journal.pntd.0004927.t003

**Table 4. Risk factors for TPPA Positivity amongst children aged 1–4.**

| Variable | Indicative Prevalence Data | Unadjusted Odds Ratio (95% CI) | Adjusted Odds Ratio[#] (95% CI) | p-value |
|---|---|---|---|---|
| **Age [years]** | 1-2: 0.87% | 1 | | |
| | 3-4: 5.8% | 2.21* (1.55-3.15) | 2.23 (1.51-3.29) | 0.001 |
| **Male** | Male: 4.0% | 1.53 (0.58-4.06) | 1.64 (0.56-4.82) | 0.33 |
| | Female: 2.7% | 1 | | |
| **Household Size [number of residents]** | ≤5: 4.0% | 0.72 (0.25-2.08) | 0.78 (0.25-2.35) | 0.62 |
| | >5: 2.9% | 1 | | |

* Increased odds associated with each one year increase in age
# Adjusted for age and gender

doi:10.1371/journal.pntd.0004927.t004

that the benefit appears to extend to 18 months without any further intervention. The WHO yaws eradication strategy [4] suggests that, following initial mass treatment, follow-up surveys with targeted treatment of cases and contacts should take place every 3–6 months. This requirement for at least biannual intervention is a potential barrier to integrating yaws eradication efforts with other NTD control programmes, including trachoma, where annual mass treatment is recommended. The absence of any active cases of yaws in these communities and the lack of a significant change in seroprevalence from 6 to 18 months after mass treatment might suggest that interventions could be spaced at yearly intervals without a significant loss of impact. Consistent with these findings we have previously reported a decline in the number of routinely reported cases of yaws in Western provinces following MDA [10]. In 2013 Western province reported 5,231 cases of yaws and this declined to 3,018 in 2014 and 2,252 cases in 2015. This routine reporting data supports our finding that the impact of MDA has been sustained up to 18 months.

Modelling studies exploring the optimal spacing of MDA are under way, and initial results suggest that such decisions are highly dependent on the basic reproductive number ($R_0$) of yaws. Where $R_0$ is low ($<1.45$) then annually spaced treatment may be sufficient to achieve interruption of transmission (M Marks–Manuscript submitted).

Of particular interest, the village-level treatment coverage was the strongest risk factor for dual-seropositivity 18 months after mass treatment. When the coverage was below 80%, the risk for seropositivity was more than 6 times greater compared to villages where coverage of greater than 90% was achieved. Consistent with these findings in older children, we noted that in villages where very high (>90%) coverage was achieved there were no seropositive children aged 1–4 years, which may indicate that transmission was interrupted in these communities. Ongoing presumed seroconversion, including in children aged 2 years or less, was documented, however, in villages with coverage below 90%. We cannot exclude the possibility that positive serology in children aged 1–4 years was due to mother-to-child transmission of syphilis. However, in the context of ongoing transmission of yaws amongst older children in these communities, we consider it likely that seropositivity amongst children aged 1–4 years does reflect ongoing transmission of yaws in this age group. These two findings emphasize the need for extremely high coverage during mass treatment. Given the difficulties that can be faced in achieving such high coverage at a programmatic level [18], innovative strategies should be considered to augment yaws eradication efforts. This might include additional school based mass treatment alongside community based approaches, or use of multiple rounds of mass treatment before transitioning to the total targeted treatment phase of the yaws eradication strategy [4].

Our data add to the literature supporting a lower dose of azithromycin (20mg/kg max 1g vs 30mg/kg max 2g) for the treatment of yaws. If a lower dose is proven effective this may help achieve programmatic synergies, especially in the Pacific where several countries have a high prevalence of yaws, and trachoma is also endemic. A WHO sponsored trial (NCT02344628) is being undertaken in Ghana and Papua New Guinea to formally compare the efficacy of these two doses in both active and latent yaws, and it is hoped that this study will provide a definitive answer on the efficacy of the lower dose of azithromycin. The prevalence of ulcers caused by *Haemophilus ducreyi* remained lower than in our pre-MDA study but has not fallen as markedly as that of yaws ulcers. We also noted a large number of ulcers of an unclear aetiology and further studies are required to better understand other causes of ulcers in a post-MDA setting.

The major limitation of our study is the observational design. A study of mass treatment in Papua New Guinea found only 44 serologically confirmed active cases in a total population of 13,166 individuals seen at six months follow-up. Given the smaller sample size of this study, we may have been underpowered to detect rare active cases following the initial round of mass treatment. A larger sample size would also have allowed more precise estimates of the seroprevalence of infection in both age groups but would probably not have altered the major findings of this study.

Our data show that the impact of a single round of mass treatment on yaws prevalence is profound, and appears to last for at least 18 months following mass treatment even in the absence of further interventions. We have demonstrated that mass treatment coverage above 90% is associated with significantly better outcomes than lower coverage. Strategies to improve coverage at all stages of yaws eradication efforts should be investigated.

## Supporting Information

**S1 Checklist. Strobe Checklist**
(DOCX)

**S1 File. Supplementary Data File**
(CSV)

## Acknowledgments

The authors would like to thank Kai-Hua Chi for her assistance with real-time PCR. The findings and conclusions in this report are those of the author(s) and do not necessarily represent the views of the Centers for Disease Control and Prevention or the World Health Organization.

## Author Contributions

**Conceived and designed the experiments:** MM OS AP CB AWS DCM.

**Performed the experiments:** MM OS EN EP GK.

**Analyzed the data:** MM CB AWS AP DCM.

**Contributed reagents/materials/analysis tools:** AP EN.

**Wrote the paper:** MM OS EP EN GK CB AP AWS DCM.

## References


1. Marks M, Solomon AW, Mabey DC. Endemic treponemal diseases. Trans. R. Soc. Trop. Med. Hyg. 2014; 108:601–607. doi: 10.1093/trstmh/tru128 PMID: 25157125
2. Mitjà O, Marks M, Konan DJP, et al. Global epidemiology of yaws: a systematic review. Lancet Glob. Health 2015; 3:e324–331. doi: 10.1016/S2214-109X(15)00011-X PMID: 26001576
3. Mitjà O, Hays R, Ipai A, et al. Single-dose azithromycin versus benzathine benzylpenicillin for treatment of yaws in children in Papua New Guinea: an open-label, non-inferiority, randomised trial. Lancet 2012; 379:342–347. doi: 10.1016/S0140-6736(11)61624-3 PMID: 22240407
4. The World Health Organisation. Eradication of yaws—the Morges Strategy. Wkly. Epidemiol. Rec. 2012; 87:189–194. PMID: 24340400
5. Chi K-H, Danavall D, Taleo F, et al. Molecular Differentiation of *Treponema pallidum* Subspecies in Skin Ulceration Clinically Suspected as Yaws in Vanuatu Using Real-Time Multiplex PCR and Serological Methods. Am. J. Trop. Med. Hyg. 2015; 92:134–138. doi: 10.4269/ajtmh.14-0459 PMID: 25404075
6. Marks M, Vahi V, Sokana O, et al. Mapping the Epidemiology of Yaws in the Solomon Islands: A Cluster Randomized Survey. Am. J. Trop. Med. Hyg. 2015; 92:129–133. doi: 10.4269/ajtmh.14-0438 PMID: 25422395
7. Mitjà O, Houinei W, Moses P, et al. Mass Treatment with Single-Dose Azithromycin for Yaws. N. Engl. J. Med. 2015; 372:703–710. doi: 10.1056/NEJMoa1408586 PMID: 25693010
8. Emerson PM, Burton MJ, Solomon AW, Bailey R, Mabey DC. The SAFE strategy for trachoma control: using operational research for policy, and implementation. Bull. World Health Organ. 2006; 84:613–619. PMID: 16917648
9. Solomon AW, Marks M, Martin DL, et al. Trachoma and Yaws: Common Ground? PLoS Negl Trop Dis 2015; 9:e0004071. doi: 10.1371/journal.pntd.0004071 PMID: 26633176
10. Marks M, Vahi V, Sokana O, et al. Impact of Community Mass Treatment with Azithromycin for Trachoma Elimination on the Prevalence of Yaws. PLoS Negl Trop Dis 2015; 9:e0003988. doi: 10.1371/journal.pntd.0003988 PMID: 26241484
11. Marks M, Mitjà O, Vestergaard LS, et al. Challenges and key research questions for yaws eradication. Lancet Infect. Dis. 2015; 15:1220–1225. doi: 10.1016/S1473-3099(15)00136-X PMID: 26362174
12. Butcher Robert, Sokana Oliver, Macleod Colin, et al. Low prevalence of conjunctival infection with Chlamydia trachomatis in a treatment-naïve trachoma-endemic region of the Solomon Islands. PLoS Negl Trop Dis. Accepted for Publication;
13. World Health Organization. Yaws: recognition booklet for communities. 2012; http://apps.who.int/iris/bitstream/10665/75360/1/9789241504096_eng.pdf?ua=1.
14. Pavluck A, Chu B, Mann Flueckiger R, Ottesen E. Electronic Data Capture Tools for Global Health Programs: Evolution of LINKS, an Android-, Web-Based System. PLoS Negl Trop Dis 2014; 8:e2654. doi: 10.1371/journal.pntd.0002654 PMID: 24722343
15. Smit PW, Vlis T van der, Mabey D, et al. The development and validation of dried blood spots for external quality assurance of syphilis serology. BMC Infect. Dis. 2013; 13:102. doi: 10.1186/1471-2334-13-102 PMID: 23442198
16. Marks M, Chi K-H, Vahi V, et al. *Haemophilus ducreyi* Associated with Skin Ulcers among Children, Solomon Islands. Emerg. Infect. Dis. 2014; 20:1705–1707. doi: 10.3201/eid2010.140573 PMID: 25271477
17. Report on 2009 Population & Housing Census. Solomon Islands Government, 2011. http://www.mof.gov.sb/Libraries/Statistics/2011_06_-_Report_on_2009_Population_Housing_Census.sflb.ashx. Accessed 27 March 2013.
18. Worrell C, Mathieu E. Drug Coverage Surveys for Neglected Tropical Diseases: 10 Years of Field Experience. Am. J. Trop. Med. Hyg. 2012; 87:216–222. doi: 10.4269/ajtmh.2012.12-0167 PMID: 22855750
